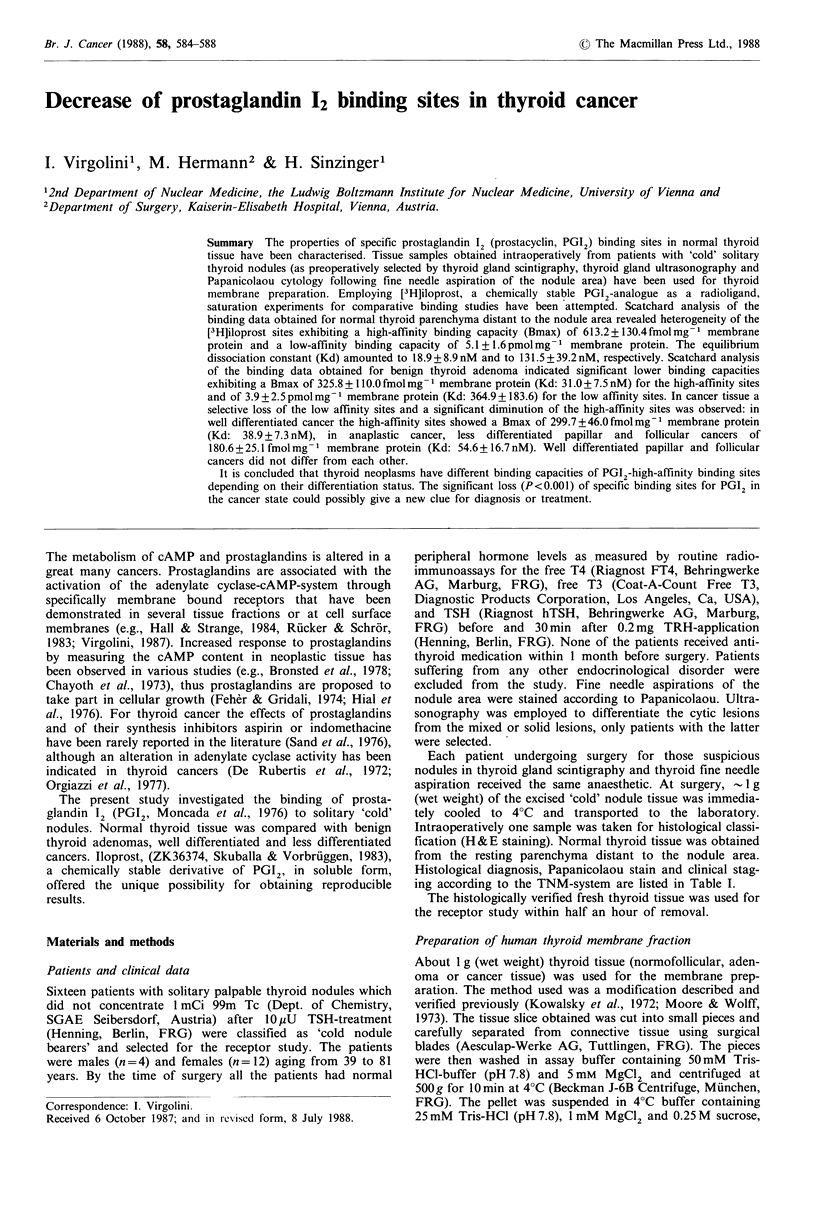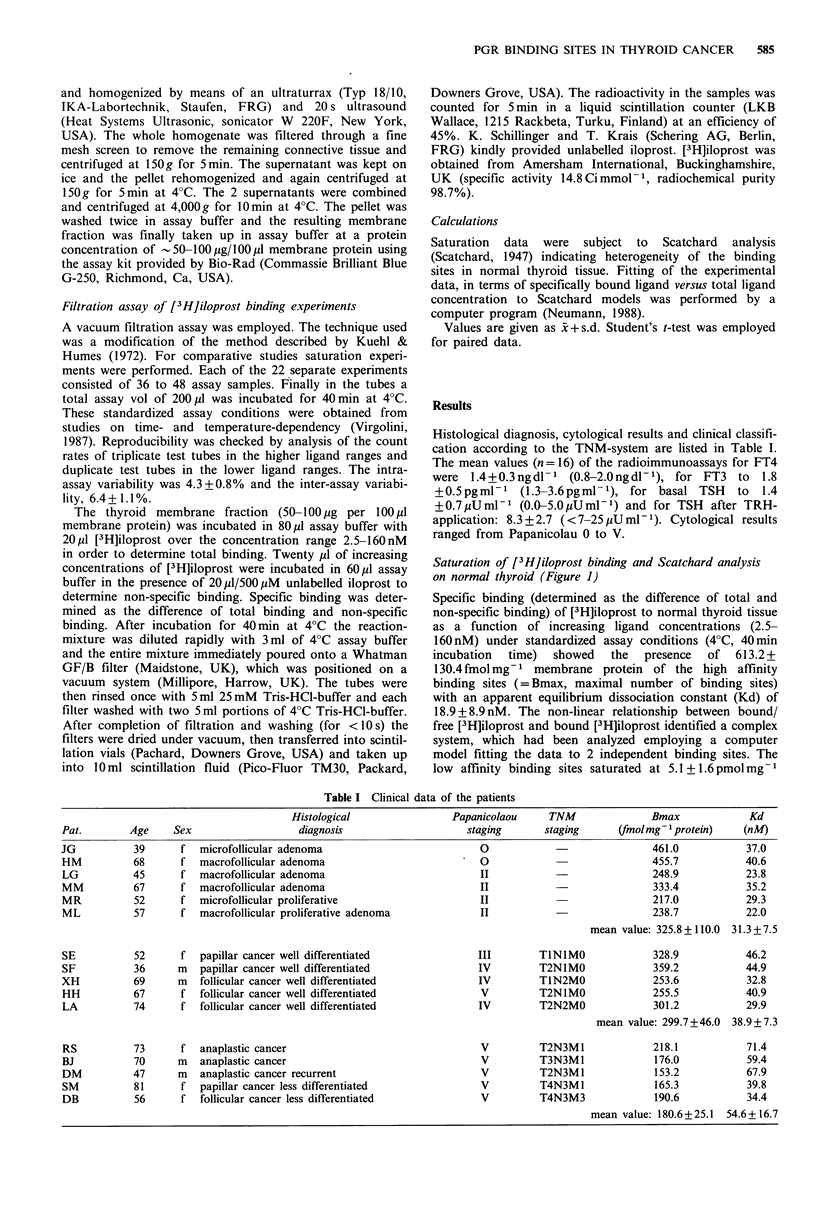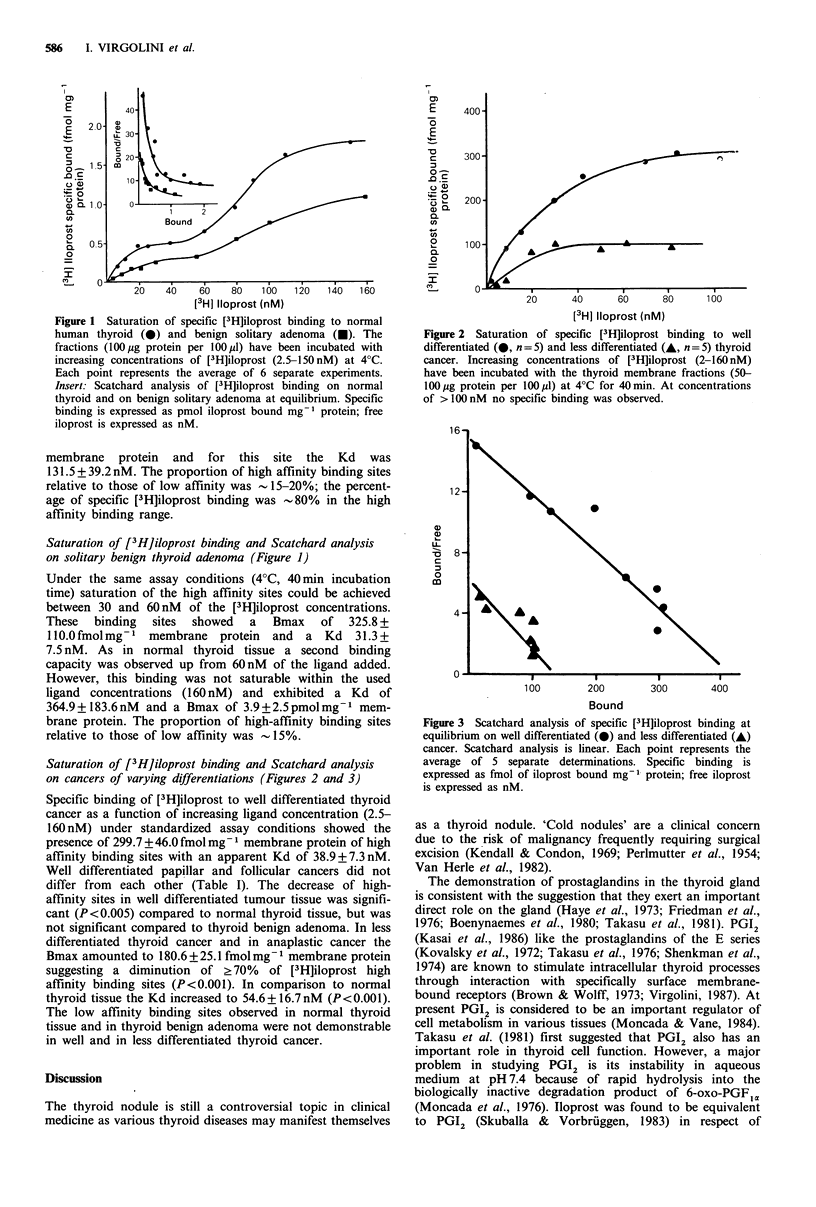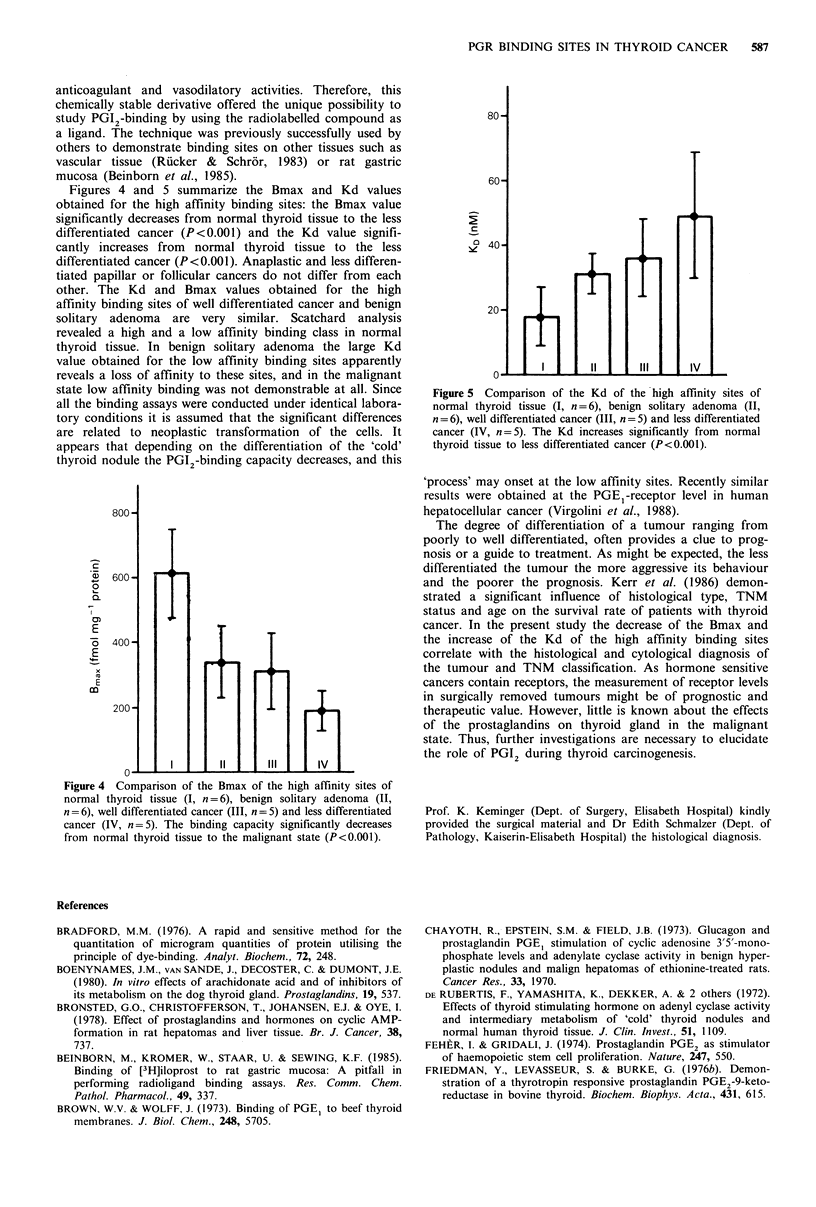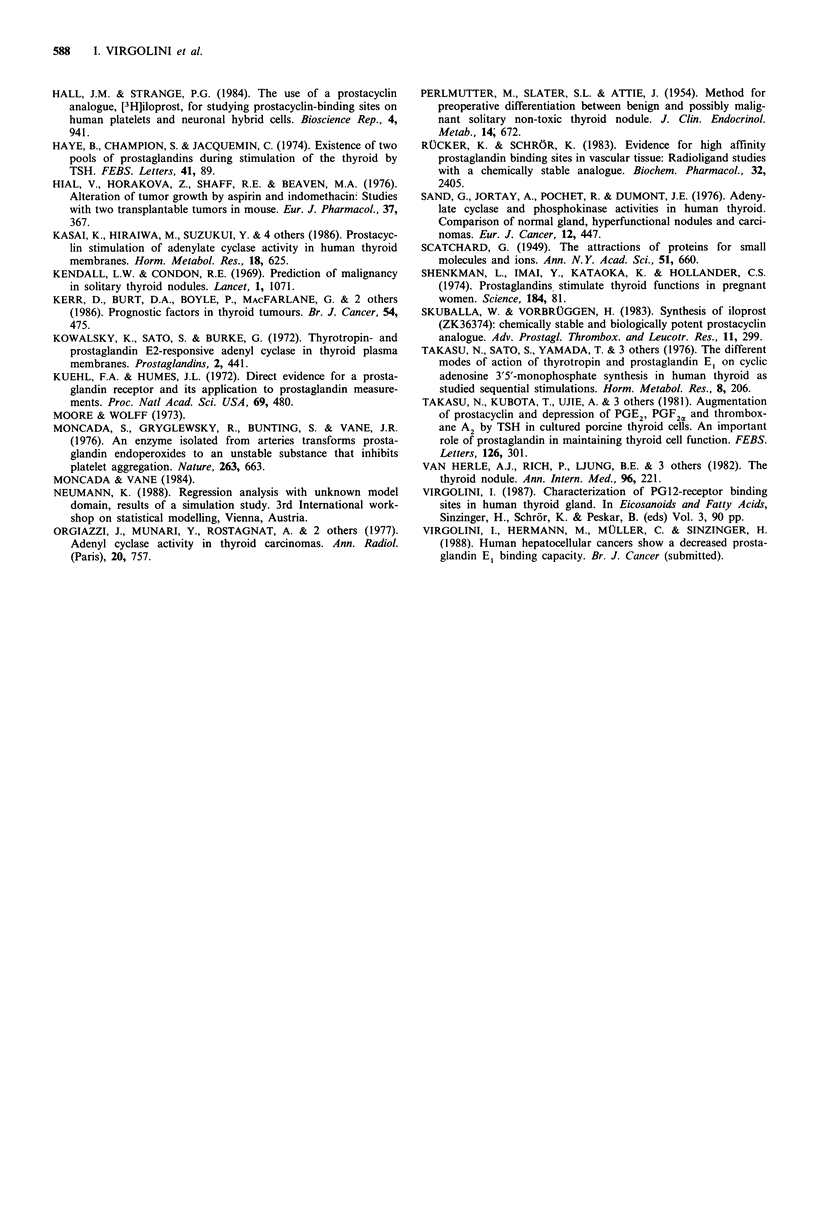# Decrease of prostaglandin I2 binding sites in thyroid cancer.

**DOI:** 10.1038/bjc.1988.264

**Published:** 1988-11

**Authors:** I. Virgolini, M. Hermann, H. Sinzinger

**Affiliations:** 2nd Department of Nuclear Medicine, Ludwig Boltzmann Institute for Nuclear Medicine, University of Vienna, Austria.

## Abstract

The properties of specific prostaglandin I2 (prostacyclin, PGI2) binding sites in normal thyroid tissue have been characterised. Tissue samples obtained intraoperatively from patients with 'cold' solitary thyroid nodules (as preoperatively selected by thyroid gland scintigraphy, thyroid gland ultrasonography and Papanicolaou cytology following fine needle aspiration of the nodule area) have been used for thyroid membrane preparation. Employing [3H]iloprost, a chemically stable PGI2-analogue as a radioligand, saturation experiments for comparative binding studies have been attempted. Scatchard analysis of the binding data obtained for normal thyroid parenchyma distant to the nodule area revealed heterogeneity of the [3H]iloprost sites exhibiting a high-affinity binding capacity (Bmax) of 613.2 +/- 130.4 fmol mg-1 membrane protein and a low-affinity binding capacity of 5.1 +/- 1.6 pmol mg-1 membrane protein. The equilibrium dissociation constant (Kd) amounted to 18.9 +/- 8.9 nM and to 131.5 +/- 39.2 nM, respectively. Scatchard analysis of the binding data obtained for benign thyroid adenoma indicated significant lower binding capacities exhibiting a Bmax of 325.8 +/- 110.0 fmol mg-1 membrane protein (Kd: 31.0 +/- 7.5 nM) for the high-affinity sites and of 3.9 +/- 2.5 pmol mg-1 membrane protein (Kd: 364.9 +/- 183.6) for the low affinity sites. In cancer tissue a selective loss of the low affinity sites and a significant diminution of the high-affinity sites was observed: in well differentiated cancer the high-affinity sites showed a Bmax of 299.7 +/- 46.0 fmol mg-1 membrane protein (Kd: 38.9 +/- 7.3 nM), in anaplastic cancer, less differentiated papillar and follicular cancers of 180.6 +/- 25.1 fmol mg-1 membrane protein (Kd: 54.6 +/- 16.7 nM). Well differentiated papillar and follicular cancers did not differ from each other.(ABSTRACT TRUNCATED AT 250 WORDS)


*Br. J. Cancer* (1988), **58**, 584–588 © The Macmillan Press Ltd., 1988

### Decrease of prostaglandin $I_2$ binding sites in thyroid cancer

I. Virgolini[1], M. Hermann[2] & H. Sinzinger[1]

[1]*2nd Department of Nuclear Medicine, the Ludwig Boltzmann Institute for Nuclear Medicine, University of Vienna and* [2]*Department of Surgery, Kaiserin-Elisabeth Hospital, Vienna, Austria.*

**Summary** The properties of specific prostaglandin $I_2$ (prostacyclin, $PGI_2$) binding sites in normal thyroid tissue have been characterised. Tissue samples obtained intraoperatively from patients with 'cold' solitary thyroid nodules (as preoperatively selected by thyroid gland scintigraphy, thyroid gland ultrasonography and Papanicolaou cytology following fine needle aspiration of the nodule area) have been used for thyroid membrane preparation. Employing [$^3$H]iloprost, a chemically stable $PGI_2$-analogue as a radioligand, saturation experiments for comparative binding studies have been attempted. Scatchard analysis of the binding data obtained for normal thyroid parenchyma distant to the nodule area revealed heterogeneity of the [$^3$H]iloprost sites exhibiting a high-affinity binding capacity (Bmax) of $613.2 \pm 130.4\,\text{fmol}\,\text{mg}^{-1}$ membrane protein and a low-affinity binding capacity of $5.1 \pm 1.6\,\text{pmol}\,\text{mg}^{-1}$ membrane protein. The equilibrium dissociation constant (Kd) amounted to $18.9 \pm 8.9$ nM and to $131.5 \pm 39.2$ nM, respectively. Scatchard analysis of the binding data obtained for benign thyroid adenoma indicated significant lower binding capacities exhibiting a Bmax of $325.8 \pm 110.0\,\text{fmol}\,\text{mg}^{-1}$ membrane protein (Kd: $31.0 \pm 7.5$ nM) for the high-affinity sites and of $3.9 \pm 2.5\,\text{pmol}\,\text{mg}^{-1}$ membrane protein (Kd: $364.9 \pm 183.6$) for the low affinity sites. In cancer tissue a selective loss of the low affinity sites and a significant diminution of the high-affinity sites was observed: in well differentiated cancer the high-affinity sites showed a Bmax of $299.7 \pm 46.0\,\text{fmol}\,\text{mg}^{-1}$ membrane protein (Kd: $38.9 \pm 7.3$ nM), in anaplastic cancer, less differentiated papillar and follicular cancers of $180.6 \pm 25.1\,\text{fmol}\,\text{mg}^{-1}$ membrane protein (Kd: $54.6 \pm 16.7$ nM). Well differentiated papillar and follicular cancers did not differ from each other.

It is concluded that thyroid neoplasms have different binding capacities of $PGI_2$-high-affinity binding sites depending on their differentiation status. The significant loss ($P < 0.001$) of specific binding sites for $PGI_2$ in the cancer state could possibly give a new clue for diagnosis or treatment.

The metabolism of cAMP and prostaglandins is altered in a great many cancers. Prostaglandins are associated with the activation of the adenylate cyclase-cAMP-system through specifically membrane bound receptors that have been demonstrated in several tissue fractions or at cell surface membranes (e.g., Hall & Strange, 1984, Rücker & Schrör, 1983; Virgolini, 1987). Increased response to prostaglandins by measuring the cAMP content in neoplastic tissue has been observed in various studies (e.g., Bronsted *et al.*, 1978; Chayoth *et al.*, 1973), thus prostaglandins are proposed to take part in cellular growth (Fehèr & Gridali, 1974; Hial *et al.*, 1976). For thyroid cancer the effects of prostaglandins and of their synthesis inhibitors aspirin or indomethacine have been rarely reported in the literature (Sand *et al.*, 1976), although an alteration in adenylate cyclase activity has been indicated in thyroid cancers (De Rubertis *et al.*, 1972; Orgiazzi *et al.*, 1977).

The present study investigated the binding of prostaglandin $I_2$ ($PGI_2$, Moncada *et al.*, 1976) to solitary 'cold' nodules. Normal thyroid tissue was compared with benign thyroid adenomas, well differentiated and less differentiated cancers. Iloprost, (ZK36374, Skuballa & Vorbrüggen, 1983), a chemically stable derivative of $PGI_2$, in soluble form, offered the unique possibility for obtaining reproducible results.

### Materials and methods

### *Patients and clinical data*

Sixteen patients with solitary palpable thyroid nodules which did not concentrate 1 mCi 99m Tc (Dept. of Chemistry, SGAE Seibersdorf, Austria) after 10 $\mu$U TSH-treatment (Henning, Berlin, FRG) were classified as 'cold nodule bearers' and selected for the receptor study. The patients were males ($n=4$) and females ($n=12$) aging from 39 to 81 years. By the time of surgery all the patients had normal peripheral hormone levels as measured by routine radioimmunoassays for the free T4 (Riagnost FT4, Behringwerke AG, Marburg, FRG), free T3 (Coat-A-Count Free T3, Diagnostic Products Corporation, Los Angeles, Ca, USA), and TSH (Riagnost hTSH, Behringwerke AG, Marburg, FRG) before and 30 min after 0.2 mg TRH-application (Henning, Berlin, FRG). None of the patients received antithyroid medication within 1 month before surgery. Patients suffering from any other endocrinological disorder were excluded from the study. Fine needle aspirations of the nodule area were stained according to Papanicolaou. Ultrasonography was employed to differentiate the cytic lesions from the mixed or solid lesions, only patients with the latter were selected.

Each patient undergoing surgery for those suspicious nodules in thyroid gland scintigraphy and thyroid fine needle aspiration received the same anaesthetic. At surgery, $\sim$1 g (wet weight) of the excised 'cold' nodule tissue was immediately cooled to 4°C and transported to the laboratory. Intraoperatively one sample was taken for histological classification (H&E staining). Normal thyroid tissue was obtained from the resting parenchyma distant to the nodule area. Histological diagnosis, Papanicolaou stain and clinical staging according to the TNM-system are listed in Table I.

The histologically verified fresh thyroid tissue was used for the receptor study within half an hour of removal.

### *Preparation of human thyroid membrane fraction*

About 1 g (wet weight) thyroid tissue (normofollicular, adenoma or cancer tissue) was used for the membrane preparation. The method used was a modification described and verified previously (Kowalsky *et al.*, 1972; Moore & Wolff, 1973). The tissue slice obtained was cut into small pieces and carefully separated from connective tissue using surgical blades (Aesculap-Werke AG, Tuttlingen, FRG). The pieces were then washed in assay buffer containing 50 mM Tris-HCl-buffer (pH 7.8) and 5 mM $MgCl_2$ and centrifuged at 500 *g* for 10 min at 4°C (Beckman J-6B Centrifuge, München, FRG). The pellet was suspended in 4°C buffer containing 25 mM Tris-HCl (pH 7.8), 1 mM $MgCl_2$ and 0.25 M sucrose,

Correspondence: I. Virgolini.
Received 6 October 1987; and in revised form, 8 July 1988.

and homogenized by means of an ultraturrax (Typ 18/10, IKA-Labortechnik, Staufen, FRG) and 20 s ultrasound (Heat Systems Ultrasonic, sonicator W 220F, New York, USA). The whole homogenate was filtered through a fine mesh screen to remove the remaining connective tissue and centrifuged at 150 *g* for 5 min. The supernatant was kept on ice and the pellet rehomogenized and again centrifuged at 150 *g* for 5 min at 4°C. The 2 supernatants were combined and centrifuged at 4,000 *g* for 10 min at 4°C. The pellet was washed twice in assay buffer and the resulting membrane fraction was finally taken up in assay buffer at a protein concentration of ~50–100 μg/100 μl membrane protein using the assay kit provided by Bio-Rad (Commassie Brilliant Blue G-250, Richmond, Ca, USA).

*Filtration assay of [³H]iloprost binding experiments*

A vacuum filtration assay was employed. The technique used was a modification of the method described by Kuehl & Humes (1972). For comparative studies saturation experiments were performed. Each of the 22 separate experiments consisted of 36 to 48 assay samples. Finally in the tubes a total assay vol of 200 μl was incubated for 40 min at 4°C. These standardized assay conditions were obtained from studies on time- and temperature-dependency (Virgolini, 1987). Reproducibility was checked by analysis of the count rates of triplicate test tubes in the higher ligand ranges and duplicate test tubes in the lower ligand ranges. The intra-assay variability was 4.3±0.8% and the inter-assay variability, 6.4±1.1%.

The thyroid membrane fraction (50–100 μg per 100 μl membrane protein) was incubated in 80 μl assay buffer with 20 μl [³H]iloprost over the concentration range 2.5–160 nM in order to determine total binding. Twenty μl of increasing concentrations of [³H]iloprost were incubated in 60 μl assay buffer in the presence of 20 μl/500 μM unlabelled iloprost to determine non-specific binding. Specific binding was determined as the difference of total binding and non-specific binding. After incubation for 40 min at 4°C the reaction-mixture was diluted rapidly with 3 ml of 4°C assay buffer and the entire mixture immediately poured onto a Whatman GF/B filter (Maidstone, UK), which was positioned on a vacuum system (Millipore, Harrow, UK). The tubes were then rinsed once with 5 ml 25 mM Tris-HCl-buffer and each filter washed with two 5 ml portions of 4°C Tris-HCl-buffer. After completion of filtration and washing (for <10 s) the filters were dried under vacuum, then transferred into scintillation vials (Pachard, Downers Grove, USA) and taken up into 10 ml scintillation fluid (Pico-Fluor TM30, Packard, Downers Grove, USA). The radioactivity in the samples was counted for 5 min in a liquid scintillation counter (LKB Wallace, 1215 Rackbeta, Turku, Finland) at an efficiency of 45%. K. Schillinger and T. Krais (Schering AG, Berlin, FRG) kindly provided unlabelled iloprost. [³H]iloprost was obtained from Amersham International, Buckinghamshire, UK (specific activity 14.8 Ci mmol$^{-1}$, radiochemical purity 98.7%).

*Calculations*

Saturation data were subject to Scatchard analysis (Scatchard, 1947) indicating heterogeneity of the binding sites in normal thyroid tissue. Fitting of the experimental data, in terms of specifically bound ligand *versus* total ligand concentration to Scatchard models was performed by a computer program (Neumann, 1988).

Values are given as $\bar{x}$+s.d. Student's *t*-test was employed for paired data.

### Results

Histological diagnosis, cytological results and clinical classification according to the TNM-system are listed in Table I. The mean values ($n=16$) of the radioimmunoassays for FT4 were 1.4±0.3 ng dl$^{-1}$ (0.8–2.0 ng dl$^{-1}$), for FT3 to 1.8 ±0.5 pg ml$^{-1}$ (1.3–3.6 pg ml$^{-1}$), for basal TSH to 1.4 ±0.7 μU ml$^{-1}$ (0.0–5.0 μU ml$^{-1}$) and for TSH after TRH-application: 8.3±2.7 (<7–25 μU ml$^{-1}$). Cytological results ranged from Papanicolau 0 to V.

*Saturation of [³H]iloprost binding and Scatchard analysis on normal thyroid (Figure 1)*

Specific binding (determined as the difference of total and non-specific binding) of [³H]iloprost to normal thyroid tissue as a function of increasing ligand concentrations (2.5–160 nM) under standardized assay conditions (4°C, 40 min incubation time) showed the presence of 613.2±130.4 fmol mg$^{-1}$ membrane protein of the high affinity binding sites (=Bmax, maximal number of binding sites) with an apparent equilibrium dissociation constant (Kd) of 18.9±8.9 nM. The non-linear relationship between bound/free [³H]iloprost and bound [³H]iloprost identified a complex system, which had been analyzed employing a computer model fitting the data to 2 independent binding sites. The low affinity binding sites saturated at 5.1±1.6 pmol mg$^{-1}$

**Table I** Clinical data of the patients

| *Pat.* | *Age* | *Sex* | *Histological diagnosis* | *Papanicolaou staging* | *TNM staging* | *Bmax (fmol mg⁻¹ protein)* | *Kd (nM)* |
|---|---|---|---|---|---|---|---|
| JG | 39 | f | microfollicular adenoma | O | — | 461.0 | 37.0 |
| HM | 68 | f | macrofollicular adenoma | O | — | 455.7 | 40.6 |
| LG | 45 | f | macrofollicular adenoma | II | — | 248.9 | 23.8 |
| MM | 67 | f | macrofollicular adenoma | II | — | 333.4 | 35.2 |
| MR | 52 | f | microfollicular proliferative | II | — | 217.0 | 29.3 |
| ML | 57 | f | macrofollicular proliferative adenoma | II | — | 238.7 | 22.0 |
| | | | | | | mean value: 325.8±110.0 | 31.3±7.5 |
| SE | 52 | f | papillar cancer well differentiated | III | T1N1M0 | 328.9 | 46.2 |
| SF | 36 | m | papillar cancer well differentiated | IV | T2N1M0 | 359.2 | 44.9 |
| XH | 69 | m | follicular cancer well differentiated | IV | T1N2M0 | 253.6 | 32.8 |
| HH | 67 | f | follicular cancer well differentiated | V | T2N1M0 | 255.5 | 40.9 |
| LA | 74 | f | follicular cancer well differentiated | IV | T2N2M0 | 301.2 | 29.9 |
| | | | | | | mean value: 299.7±46.0 | 38.9±7.3 |
| RS | 73 | f | anaplastic cancer | V | T2N3M1 | 218.1 | 71.4 |
| BJ | 70 | m | anaplastic cancer | V | T3N3M1 | 176.0 | 59.4 |
| DM | 47 | m | anaplastic cancer recurrent | V | T2N3M1 | 153.2 | 67.9 |
| SM | 81 | f | papillar cancer less differentiated | V | T4N3M1 | 165.3 | 39.8 |
| DB | 56 | f | follicular cancer less differentiated | V | T4N3M3 | 190.6 | 34.4 |
| | | | | | | mean value: 180.6±25.1 | 54.6±16.7 |

**Figure 1** Saturation of specific [$^3$H]iloprost binding to normal human thyroid (●) and benign solitary adenoma (■). The fractions (100 μg protein per 100 μl) have been incubated with increasing concentrations of [$^3$H]iloprost (2.5–150 nM) at 4°C. Each point represents the average of 6 separate experiments. *Insert:* Scatchard analysis of [$^3$H]iloprost binding on normal thyroid and on benign solitary adenoma at equilibrium. Specific binding is expressed as pmol iloprost bound $mg^{-1}$ protein; free iloprost is expressed as nM.

**Figure 2** Saturation of specific [$^3$H]iloprost binding to well differentiated (●, $n=5$) and less differentiated (▲, $n=5$) thyroid cancer. Increasing concentrations of [$^3$H]iloprost (2–160 nM) have been incubated with the thyroid membrane fractions (50–100 μg protein per 100 μl) at 4°C for 40 min. At concentrations of >100 nM no specific binding was observed.

**Figure 3** Scatchard analysis of specific [$^3$H]iloprost binding at equilibrium on well differentiated (●) and less differentiated (▲) cancer. Scatchard analysis is linear. Each point represents the average of 5 separate determinations. Specific binding is expressed as fmol of iloprost bound $mg^{-1}$ protein; free iloprost is expressed as nM.

membrane protein and for this site the Kd was 131.5 ± 39.2 nM. The proportion of high affinity binding sites relative to those of low affinity was ~15–20%; the percentage of specific [$^3$H]iloprost binding was ~80% in the high affinity binding range.

*Saturation of [$^3$H]iloprost binding and Scatchard analysis on solitary benign thyroid adenoma (Figure 1)*

Under the same assay conditions (4°C, 40 min incubation time) saturation of the high affinity sites could be achieved between 30 and 60 nM of the [$^3$H]iloprost concentrations. These binding sites showed a Bmax of 325.8 ± 110.0 fmol $mg^{-1}$ membrane protein and a Kd 31.3 ± 7.5 nM. As in normal thyroid tissue a second binding capacity was observed up from 60 nM of the ligand added. However, this binding was not saturable within the used ligand concentrations (160 nM) and exhibited a Kd of 364.9 ± 183.6 nM and a Bmax of 3.9 ± 2.5 pmol $mg^{-1}$ membrane protein. The proportion of high-affinity binding sites relative to those of low affinity was ~15%.

*Saturation of [$^3$H]iloprost binding and Scatchard analysis on cancers of varying differentiations (Figures 2 and 3)*

Specific binding of [$^3$H]iloprost to well differentiated thyroid cancer as a function of increasing ligand concentration (2.5–160 nM) under standardized assay conditions showed the presence of 299.7 ± 46.0 fmol $mg^{-1}$ membrane protein of high affinity binding sites with an apparent Kd of 38.9 ± 7.3 nM. Well differentiated papillar and follicular cancers did not differ from each other (Table I). The decrease of high-affinity sites in well differentiated tumour tissue was significant ($P<0.005$) compared to normal thyroid tissue, but was not significant compared to thyroid benign adenoma. In less differentiated thyroid cancer and in anaplastic cancer the Bmax amounted to 180.6 ± 25.1 fmol $mg^{-1}$ membrane protein suggesting a diminution of ≥70% of [$^3$H]iloprost high affinity binding sites ($P<0.001$). In comparison to normal thyroid tissue the Kd increased to 54.6 ± 16.7 nM ($P<0.001$). The low affinity binding sites observed in normal thyroid tissue and in thyroid benign adenoma were not demonstrable in well and in less differentiated thyroid cancer.

### Discussion

The thyroid nodule is still a controversial topic in clinical medicine as various thyroid diseases may manifest themselves as a thyroid nodule. 'Cold nodules' are a clinical concern due to the risk of malignancy frequently requiring surgical excision (Kendall & Condon, 1969; Perlmutter *et al.*, 1954; Van Herle *et al.*, 1982).

The demonstration of prostaglandins in the thyroid gland is consistent with the suggestion that they exert an important direct role on the gland (Haye *et al.*, 1973; Friedman *et al.*, 1976; Boenynaemes *et al.*, 1980; Takasu *et al.*, 1981). $PGI_2$ (Kasai *et al.*, 1986) like the prostaglandins of the E series (Kovalsky *et al.*, 1972; Takasu *et al.*, 1976; Shenkman *et al.*, 1974) are known to stimulate intracellular thyroid processes through interaction with specifically surface membrane-bound receptors (Brown & Wolff, 1973; Virgolini, 1987). At present $PGI_2$ is considered to be an important regulator of cell metabolism in various tissues (Moncada & Vane, 1984). Takasu *et al.* (1981) first suggested that $PGI_2$ also has an important role in thyroid cell function. However, a major problem in studying $PGI_2$ is its instability in aqueous medium at pH 7.4 because of rapid hydrolysis into the biologically inactive degradation product of 6-oxo-$PGF_{1\alpha}$ (Moncada *et al.*, 1976). Iloprost was found to be equivalent to $PGI_2$ (Skuballa & Vorbrüggen, 1983) in respect of anticoagulant and vasodilatory activities. Therefore, this chemically stable derivative offered the unique possibility to study $PGI_2$-binding by using the radiolabelled compound as a ligand. The technique was previously successfully used by others to demonstrate binding sites on other tissues such as vascular tissue (Rücker & Schrör, 1983) or rat gastric mucosa (Beinborn *et al.*, 1985).

Figures 4 and 5 summarize the Bmax and Kd values obtained for the high affinity binding sites: the Bmax value significantly decreases from normal thyroid tissue to the less differentiated cancer ($P<0.001$) and the Kd value significantly increases from normal thyroid tissue to the less differentiated cancer ($P<0.001$). Anaplastic and less differentiated papillar or follicular cancers do not differ from each other. The Kd and Bmax values obtained for the high affinity binding sites of well differentiated cancer and benign solitary adenoma are very similar. Scatchard analysis revealed a high and a low affinity binding class in normal thyroid tissue. In benign solitary adenoma the large Kd value obtained for the low affinity binding sites apparently reveals a loss of affinity to these sites, and in the malignant state low affinity binding was not demonstrable at all. Since all the binding assays were conducted under identical laboratory conditions it is assumed that the significant differences are related to neoplastic transformation of the cells. It appears that depending on the differentiation of the 'cold' thyroid nodule the $PGI_2$-binding capacity decreases, and this

**Figure 4** Comparison of the Bmax of the high affinity sites of normal thyroid tissue (I, $n=6$), benign solitary adenoma (II, $n=6$), well differentiated cancer (III, $n=5$) and less differentiated cancer (IV, $n=5$). The binding capacity significantly decreases from normal thyroid tissue to the malignant state ($P<0.001$).

**Figure 5** Comparison of the Kd of the high affinity sites of normal thyroid tissue (I, $n=6$), benign solitary adenoma (II, $n=6$), well differentiated cancer (III, $n=5$) and less differentiated cancer (IV, $n=5$). The Kd increases significantly from normal thyroid tissue to less differentiated cancer ($P<0.001$).

'process' may onset at the low affinity sites. Recently similar results were obtained at the $PGE_1$-receptor level in human hepatocellular cancer (Virgolini *et al.*, 1988).

The degree of differentiation of a tumour ranging from poorly to well differentiated, often provides a clue to prognosis or a guide to treatment. As might be expected, the less differentiated the tumour the more aggressive its behaviour and the poorer the prognosis. Kerr *et al.* (1986) demonstrated a significant influence of histological type, TNM status and age on the survival rate of patients with thyroid cancer. In the present study the decrease of the Bmax and the increase of the Kd of the high affinity binding sites correlate with the histological and cytological diagnosis of the tumour and TNM classification. As hormone sensitive cancers contain receptors, the measurement of receptor levels in surgically removed tumours might be of prognostic and therapeutic value. However, little is known about the effects of the prostaglandins on thyroid gland in the malignant state. Thus, further investigations are necessary to elucidate the role of $PGI_2$ during thyroid carcinogenesis.

Prof. K. Keminger (Dept. of Surgery, Elisabeth Hospital) kindly provided the surgical material and Dr Edith Schmalzer (Dept. of Pathology, Kaiserin-Elisabeth Hospital) the histological diagnosis.

### References

BRADFORD, M.M. (1976). A rapid and sensitive method for the quantitation of microgram quantities of protein utilising the principle of dye-binding. *Analyt. Biochem.*, **72**, 248.

BOENYNAMES, J.M., VAN SANDE, J., DECOSTER, C. & DUMONT, J.E. (1980). *In vitro* effects of arachidonate acid and of inhibitors of its metabolism on the dog thyroid gland. *Prostaglandins*, **19**, 537.

BRONSTED, G.O., CHRISTOFFERSON, T., JOHANSEN, E.J. & OYE, I. (1978). Effect of prostaglandins and hormones on cyclic AMP-formation in rat hepatomas and liver tissue. *Br. J. Cancer*, **38**, 737.

BEINBORN, M., KROMER, W., STAAR, U. & SEWING, K.F. (1985). Binding of [³H]iloprost to rat gastric mucosa: A pitfall in performing radioligand binding assays. *Res. Comm. Chem. Pathol. Pharmacol.*, **49**, 337.

BROWN, W.V. & WOLFF, J. (1973). Binding of $PGE_1$ to beef thyroid membranes. *J. Biol. Chem.*, **248**, 5705.

CHAYOTH, R., EPSTEIN, S.M. & FIELD, J.B. (1973). Glucagon and prostaglandin $PGE_1$ stimulation of cyclic adenosine 3′5′-monophosphate levels and adenylate cyclase activity in benign hyperplastic nodules and malign hepatomas of ethionine-treated rats. *Cancer Res.*, **33**, 1970.

DE RUBERTIS, F., YAMASHITA, K., DEKKER, A. & 2 others (1972). Effects of thyroid stimulating hormone on adenyl cyclase activity and intermediary metabolism of 'cold' thyroid nodules and normal human thyroid tissue. *J. Clin. Invest.*, **51**, 1109.

FEHÈR, I. & GRIDALI, J. (1974). Prostaglandin $PGE_2$ as stimulator of haemopoietic stem cell proliferation. *Nature*, **247**, 550.

FRIEDMAN, Y., LEVASSEUR, S. & BURKE, G. (1976*b*). Demonstration of a thyrotropin responsive prostaglandin $PGE_2$-9-keto-reductase in bovine thyroid. *Biochem. Biophys. Acta.*, **431**, 615.

HALL, J.M. & STRANGE, P.G. (1984). The use of a prostacyclin analogue, [$^3$H]iloprost, for studying prostacyclin-binding sites on human platelets and neuronal hybrid cells. *Bioscience Rep.*, **4**, 941.

HAYE, B., CHAMPION, S. & JACQUEMIN, C. (1974). Existence of two pools of prostaglandins during stimulation of the thyroid by TSH. *FEBS. Letters*, **41**, 89.

HIAL, V., HORAKOVA, Z., SHAFF, R.E. & BEAVEN, M.A. (1976). Alteration of tumor growth by aspirin and indomethacin: Studies with two transplantable tumors in mouse. *Eur. J. Pharmacol.*, **37**, 367.

KASAI, K., HIRAIWA, M., SUZUKUI, Y. & 4 others (1986). Prostacyclin stimulation of adenylate cyclase activity in human thyroid membranes. *Horm. Metabol. Res.*, **18**, 625.

KENDALL, L.W. & CONDON, R.E. (1969). Prediction of malignancy in solitary thyroid nodules. *Lancet*, **1**, 1071.

KERR, D., BURT, D.A., BOYLE, P., MACFARLANE, G. & 2 others (1986). Prognostic factors in thyroid tumours. *Br. J. Cancer*, **54**, 475.

KOWALSKY, K., SATO, S. & BURKE, G. (1972). Thyrotropin- and prostaglandin E2-responsive adenyl cyclase in thyroid plasma membranes. *Prostaglandins*, **2**, 441.

KUEHL, F.A. & HUMES, J.L. (1972). Direct evidence for a prostaglandin receptor and its application to prostaglandin measurements. *Proc. Natl Acad. Sci. USA*, **69**, 480.

MOORE & WOLFF (1973).

MONCADA, S., GRYGLEWSKY, R., BUNTING, S. & VANE, J.R. (1976). An enzyme isolated from arteries transforms prostaglandin endoperoxides to an unstable substance that inhibits platelet aggregation. *Nature*, **263**, 663.

MONCADA & VANE (1984).

NEUMANN, K. (1988). Regression analysis with unknown model domain, results of a simulation study. 3rd International workshop on statistical modelling, Vienna, Austria.

ORGIAZZI, J., MUNARI, Y., ROSTAGNAT, A. & 2 others (1977). Adenyl cyclase activity in thyroid carcinomas. *Ann. Radiol.* (Paris), **20**, 757.

PERLMUTTER, M., SLATER, S.L. & ATTIE, J. (1954). Method for preoperative differentiation between benign and possibly malignant solitary non-toxic thyroid nodule. *J. Clin. Endocrinol. Metab.*, **14**, 672.

RÜCKER, K. & SCHRÖR, K. (1983). Evidence for high affinity prostaglandin binding sites in vascular tissue: Radioligand studies with a chemically stable analogue. *Biochem. Pharmacol.*, **32**, 2405.

SAND, G., JORTAY, A., POCHET, R. & DUMONT, J.E. (1976). Adenylate cyclase and phosphokinase activities in human thyroid. Comparison of normal gland, hyperfunctional nodules and carcinomas. *Eur. J. Cancer*, **12**, 447.

SCATCHARD, G. (1949). The attractions of proteins for small molecules and ions. *Ann. N.Y. Acad. Sci.*, **51**, 660.

SHENKMAN, L., IMAI, Y., KATAOKA, K. & HOLLANDER, C.S. (1974). Prostaglandins stimulate thyroid functions in pregnant women. *Science*, **184**, 81.

SKUBALLA, W. & VORBRÜGGEN, H. (1983). Synthesis of iloprost (ZK36374): chemically stable and biologically potent prostacyclin analogue. *Adv. Prostagl. Thrombox. and Leucotr. Res.*, **11**, 299.

TAKASU, N., SATO, S., YAMADA, T. & 3 others (1976). The different modes of action of thyrotropin and prostaglandin $E_1$ on cyclic adenosine 3′5′-monophosphate synthesis in human thyroid as studied sequential stimulations. *Horm. Metabol. Res.*, **8**, 206.

TAKASU, N., KUBOTA, T., UJIE, A. & 3 others (1981). Augmentation of prostacyclin and depression of $PGE_2$, $PGF_{2\alpha}$ and thromboxane $A_2$ by TSH in cultured porcine thyroid cells. An important role of prostaglandin in maintaining thyroid cell function. *FEBS. Letters*, **126**, 301.

VAN HERLE, A.J., RICH, P., LJUNG, B.E. & 3 others (1982). The thyroid nodule. *Ann. Intern. Med.*, **96**, 221.

VIRGOLINI, I. (1987). Characterization of PG12-receptor binding sites in human thyroid gland. In *Eicosanoids and Fatty Acids*, Sinzinger, H., Schrör, K. & Peskar, B. (eds) Vol. 3, 90 pp.

VIRGOLINI, I., HERMANN, M., MÜLLER, C. & SINZINGER, H. (1988). Human hepatocellular cancers show a decreased prostaglandin $E_1$ binding capacity. *Br. J. Cancer* (submitted).